# Macular arteritis in an HIV-infected patient improving with adherence to antiretroviral therapy

**DOI:** 10.1016/j.jdcr.2020.06.017

**Published:** 2020-06-14

**Authors:** Elisabeth Boddé, Jeffrey Damman, Martijn B.A. van Doorn

**Affiliations:** a Department of Dermatology and Venereology, Erasmus MC, Rotterdam, the Netherlands; b Department of Pathology, Erasmus MC, Rotterdam, the Netherlands

## Introduction

Macular arteritis is a cutaneous vasculitis characterized by asymptomatic erythematous or hyperpigmented macules on clinical examination and by the histopathologic hallmarks of a lymphocyte-predominant small- to medium-sized vessel arteritis.

Macular arteritis mostly affects middle-aged women, usually from North African or Asian countries.^1^^,^^2^ The lower extremities are predominantly involved, and besides minimal pruritus, in most cases the skin lesions are asymptomatic.^3^

## Case report

A 33-year-old HIV-positive female patient presented at our outpatient clinic, with a 6-month history of progressive brown macules on her arms, legs, and abdomen. Besides incidental complaints of slight itching, the skin lesions were asymptomatic. She had previously been treated with topical steroids, without any effect. Subsequently, she was treated with combination antiretroviral therapy, consisting of dolutegravir and emtricitabine/tenofovir disoproxil.

**Fig 1:**
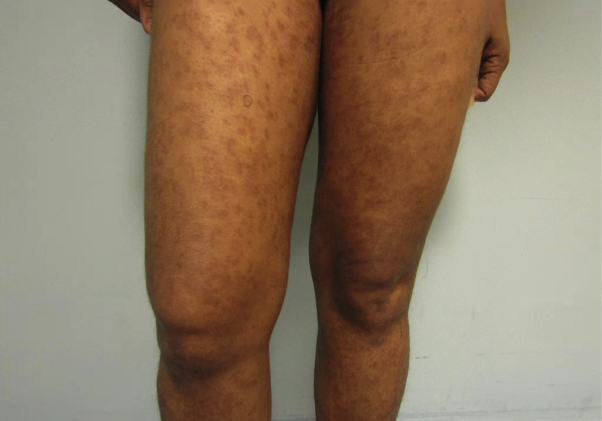
Clinical photograph of the patient.

On physical examination, we observed numerous ill-defined hyperpigmented macules on her arms, legs, and abdomen, some of which were slightly erythematous (Fig 1). The skin lesions were predominantly macular, but some exhibited slight induration.

**Fig 2:**
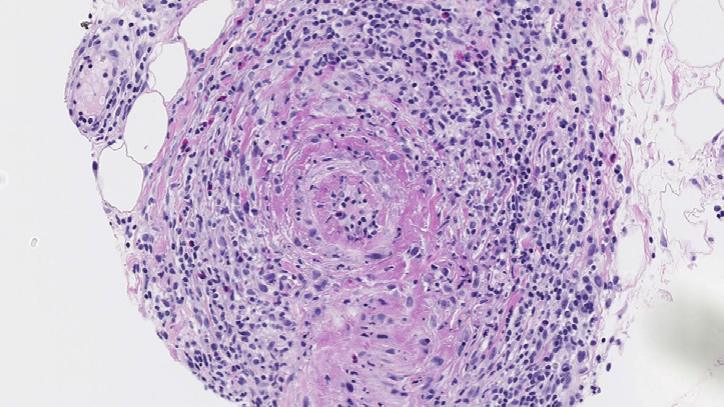
Subcutaneous small- to medium-sized arteritis showing a concentric ring of fibrin deposition primary localized to the intima but with extension into the medial wall. There is endothelial cell swelling and lymphocytic endothelialitis. Also note the extensive (peri)adventitial infiltrate composed of lymphocytes, histiocytes, plasma cells, and eosinophils but without neutrophils.

**Fig 3:**
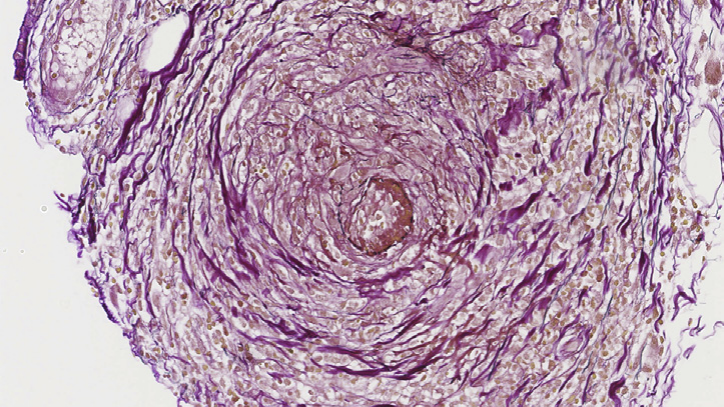
An elastin stain revealed an almost intact internal elastic lamina.

Histopathologic examination revealed a small- to medium-sized artery with transmural influx of lymphocytes and prominent concentric intimal fibrinoid necrosis (Fig 2). An elastin stain showed an intact internal elastic lamina as proof of an affected artery (Fig 3). In addition, perivascular adventitial inflammation was noted, with presence of lymphocytes, eosinophils, and few plasma cells. After clinicopathologic correlation, we made the diagnosis of macular arteritis.

The patient admitted having poor adherence to the antiretroviral therapy, which was confirmed by a reduced CD4 T lymphocyte count. By intensifying surveillance through home care, her treatment adherence improved, which eventually led to the normalization of her CD4 T lymphocyte count. Subsequently, the skin lesions faded and eventually disappeared.

## Discussion

A total of 50 cases of macular arteritis have been described in the literature.^4^ To our knowledge, only 1 case similar to ours has been reported previously, describing macular arteritis in an African woman with HIV and hepatitis B coinfection.^5^ HIV patients with small- to medium-vessel vasculitis that improved after antiretroviral therapy have been described previously in the literature. These studies included patients with intracerebral and intra-abdominal vasculitis,^6^, ^7^, ^8^ as well as skin lesions.^9^, ^10^, ^11^, ^12^, ^13^ Although vasculitis in these patients also resolved after antiviral therapy, in contrast to our case, none of these patients presented with macular skin lesions.

The estimated incidence of vasculitis in HIV-positive patients is 1% and includes all types of vasculitis (small-, medium-, and large-vessel vasculitis). Different mechanisms of different types of vasculitis in HIV patients have been identified, such as the direct invasion of vascular tissue by HIV particles. An indirect effect of HIV infection is the elevation of CD8 T cells in active HIV infections, which causes increased release of growth factors, cytokines, adhesion molecules, superantigens, and immunocomplexes.^14^ In macular arteritis, vasculitis is characterized by an endarteritic endotheliopathy through complement and type 1 interferon activation lacking transmural fibrinoid necrosis, with preservation of the vascular lamina. Macular arteritis is clinically expressed by limited cutaneous manifestations with chronic indolent course and absence of progression to systemic disease.

There has been debate in the literature about whether macular arteritis represents an indolent nonnodular variant of cutaneous polyarteritis nodosa or a distinct entity.^4^^,^^15^, ^16^, ^17^, ^18^, ^19^, ^20^, ^21^

Cutaneous polyarteritis nodosa can classically present with tender dermal nodules, palpable purpura, and ulcers, while macular arteritis shows features of pigmented macules, livedo racemosa, or both, as illustrated in the current case. Although cutaneous polyarteritis nodosa and macular arteritis show distinct clinical presentations, it is hypothesized that macular arteritis could represent an early stage in the spectrum of cutaneous polyarteritis nodosa. An additional entity that fueled this discussion is the introduction of the term “lymphocytic thrombophilic arteritis.” Patients described with lymphocytic thrombophilic arteritis show clinical features in between those of macular arteritis and cutaneous polyarteritis nodosa, with pigmented macules on the one hand and nodules on the other.^21^ The authors who introduced the term suggested replacing macular arteritis with lymphocytic thrombophilic arteritis, because in their opinion the latter describes the pathophysiology of the disease more accurately. Others, however, have suggested reserving lymphocytic thrombophilic arteritis for presentation with papules and macular arteritis purely for the macular variant. They hypothesized a spectrum of disease within macular arteritis, lymphocytic thrombophilic arteritis, and cutaneous polyarteritis nodosa.^16^ In contrast, a recent systematic review concluded that macular arteritis is distinct from cutaneous polyarteritis nodosa primarily according to the indolent stable course and lack of systemic progression of macular arteritis versus cutaneous polyarteritis nodosa. Although the diseases are still under debate, the same systematic review and most other studies now agree that macular arteritis and lymphocytic thrombophilic arteritis are identical entities.^19^

On histopathology, cutaneous polyarteritis nodosa predominantly exhibits a transmural neutrophilic vasculitis with destruction of the internal elastic lamina, although subacute or reparative phases can manifest as a lymphocytic vasculitis. Because cases described as lymphocytic thrombophilic arteritis/macular arteritis always present with a lymphocytic vasculitis, clinicopathologic correlation is essential.

In line with our case, skin biopsies of lymphocytic thrombophilic arteritis/macular arteritis have shown dominant intimal concentric fibrin deposition and a preserved internal elastic lamina. Therefore, it has been suggested that lymphocytic thrombophilic arteritis/macular arteritis, in contrast to cutaneous polyarteritis nodosa, should be classified as a lymphohistiocytic thrombophilic endovasculitis (Fig 3).^22^ Although others have argued that a preserved internal elastic lamina can also be observed in early stages of cutaneous polyarteritis nodosa, the extensive, transmural, and above all lymphohistiocytic (and not neutrophilic) inflammation, as observed in our case, argues against this. The hypothesis of a thrombophilic endovasculitis in lymphocytic thrombophilic arteritis/macular arteritis could also play a role in our case because hypercoagulability can occur in an active HIV infection; however, in our case coagulability tests were not performed.^23^

Discriminating between both entities is of value because response to treatment is substantially different. Flares of cutaneous polyarteritis nodosa are treated with high-dosage systemic glucocorticoids. Once disease control is established, a glucocorticoid-sparing agent can be initiated, such as dapsone, colchicine, or hydroxychloroquine. This, in contrast to lymphocytic thrombophilic arteritis/macular arteritis, which has a much more indolent character and has been shown to be very treatment resistant. To our knowledge, there have been no reported cases of patients with lymphocytic thrombophilic arteritis/macular arteritis with progression to systemic vasculitis.^19^^,^^20^

The diagnosis of macular arteritis can be made for a patient with macular skin lesions and the histologic hallmarks of lymphohistiocytic thrombophilic endovasculitis. In this patient, macular arteritis/lymphocytic thrombophilic arteritis significantly improved with adherence to antiretroviral therapy.
